# Potential of caveolae in the therapy of cardiovascular and neurological diseases

**DOI:** 10.3389/fphys.2014.00370

**Published:** 2014-09-30

**Authors:** Gemma Navarro, Dasiel O. Borroto-Escuela, Kjell Fuxe, Rafael Franco

**Affiliations:** ^1^Departament de Bioquímica i Biologia Molecular, Facultat de Biologia, Universitat de BarcelonaBarcelona, Spain; ^2^Department of Neuroscience, Karolinska InstitutetStockholm, Sweden

**Keywords:** arrhythmia, dementia, gene therapy, GPCR, ischemia reperfusion, neurodegeneration, Parkinson's disease, stem cell

## Abstract

Caveolae are membrane micro-domains enriched in cholesterol, sphingolipids and caveolins, which are transmembrane proteins with a hairpin-like structure. Caveolae participate in receptor-mediated trafficking of cell surface receptors and receptor-mediated signaling. Furthermore, caveolae participate in clathrin-independent endocytosis of membrane receptors. On the one hand, caveolins are involved in vascular and cardiac dysfunction. Also, neurological abnormalities in caveolin-1 knockout mice and a link between caveolin-1 gene haplotypes and neurodegenerative diseases have been reported. The aim of this article is to present the rationale for considering caveolae as potential targets in cardiovascular and neurological diseases.

## Introduction

Caveolins are a family of proteins with a hairpin-like structure. This structure is necessary to form unique membrane micro-domains known as caveolae. To our knowledge Yamada (1955) first identified caveolae in gallbladder epithelium. The author observed: “*The free cell surface between microvilli shows larger cave-like depressions, likewise representing caveolae intracellulares, containing a dense material*.” Twenty years later, Dulhunty and Franzini-Armstrong (1975) provided a detailed account of the appearance of caveolae using freeze-fracture replicas of the cell surface membrane of frog skeletal muscles. They defined the structure as: “*elliptical invaginations of the plasmalemma which open to the outside by a narrow ‘neck’ of approximately 20 nm*.” Rothberg et al. (1992) identified and named as caveolin the key component in such membrane micro-domains. Caveolins are a family of proteins with similar structure and, to date, three members have been identified. The first member, VIP21-caveolin or caveolin-1, is widely expressed in tissues, especially in adipocytes, fibroblasts, epithelial cells and vascular endothelial cells; caveolin-2 interacts with and is co-expressed with caveolin-1; and, M-caveolin or caveolin-3, is mainly expressed in striated (skeletal and smooth) muscle myocytes (Scherer et al., 1996; Tang et al., 1996; Way and Parton, 1996; see Gratton et al., 2004 and Gazzerro et al., 2010 for review). Caveolae are enriched in cholesterol and sphingolipids (Severs, 1981; Rothberg et al., 1990; Parton, 1994) and the high content in cholesterol allows manipulating the system using sterol-binding agents (e.g., methyl-β-cyclodextrin or filipin). These compounds have been indeed invaluable to study the physiological role of caveolae. Vertical domains that usually span both cell membrane leaflets and are enriched in cholesterol and sphingolipids are known as lipids rafts. Caveolae are considered lipid rafts in as much as they contain caveolins.

The two most obvious roles of caveolae are to recruit membrane proteins at specific membrane domains and to participate in protein internalization (recently reviewed in Shvets et al., 2014). Also they can regulate numerous enzyme activities, including that of adenylate cyclase (AC), eNitric oxide synthase (eNOS), and several kinases and serine/threonine phosphatases (Toya et al., 1998; Carman et al., 1999; Razani and Lisanti, 2001; Hnasko and Lisanti, 2003). Hence, caveolins/caveolae are not just organizers or scaffolds that localize signaling proteins but regulators of important cell events. This perspective article focuses on the therapeutic potential of targeting caveolins/caveolae in cardiovascular and neurological diseases.

## Caveolae and cardiovascular diseases

Altered endothelium appears as a common factor in a variety of serious diseases (Berman et al., 1990; Harrison, 1991; Lerman and Burnett, 1992; Quyyumi, 1998; Vincent et al., 2011; Salmon and Satchell, 2012). Caveolins and caveolae are very relevant to maintain the endothelial cell membrane integrity in both structure and function (Williams and Lisanti, 2004; Yuan and Rigor, 2010). Atherosclerosis, a frequent disease in Western societies, is due to deposition of cholesterol-rich lipoproteins in the endothelium of blood vessels. Transcytosis, which is a vesicle-mediated mechanism of transcellular transport of molecules, is very important to remove lipid deposits and to avoid endothelial activation and vessel occlusion. Recent evidence shows that caveolin-1 and caveolae are involved in metabolic switching, endothelial transcytosis and regulate vascular inflammation (Pavlides et al., 2014; Shiroto et al., 2014).

Often, heart abnormalities course with abnormal calcium handling, for instance, in atrial fibrillation (Hove-Madsen et al., 2004) and hypertrophy (Gwathmey and Morgan, 1985; Cuneo and Grassi de Gende, 1988). Phospholipase C and hetero-trimeric Gq proteins regulate intracellular calcium concentrations. In an elegant study Guo et al. (2011) identified Gq proteins in caveolae and reported that adult caveolin-3-containing ventricular cardiomyocytes show oscillating Ca^2+^ waves that are extinguished by blocking the interaction between caveolin-3 and the α subunit of Gq proteins. Therefore, caveolin-3 is directly involved in regulating contractility and may be a target for heart hypertrophy.

Mutations in the caveolin-1 gene and decreased expression of caveolin-1 have been identified in patients with pulmonary arterial hypertension (Desai, 2012), a disease with high morbidity. Caveolin-1 null mice display a marked reduction in life span due to a combination of cardiac hypertrophy, pulmonary fibrosis and pulmonary hypertension (Park et al., 2003). Interestingly, double KO mice for caveolin-1 and the myocyte-selective subtype, caveolin-3, are viable but display severe cardiomyopathy (Park et al., 2002). Although Feiner et al. (2011) could not demonstrate significantly different levels of caveolin-3 in failing hearts, a significant correlation existed in human failing hearts between levels of caveolin-3 and Ca^2+^-ATPase, a marker of the heart-failure phenotype. To our knowledge, Fujimoto in (1993) was the first to identify an ion pump (Ca^2+^) in caveolae. Since then several studies have confirmed a link between caveolins/caveolae and ion carriers. In cardiomyocytes the Na^+^/Ca^2+^ exchanger is very important for heart functionality. The exchanger may interact with caveolin-1 but may also form macromolecular complexes with caveolin-3 and annexin A5 (Bossuyt et al., 2002a,b). In left ventricular myocardial samples from human failing hearts the annexin A5-interaction site in the exchanger is not accessible and the interaction between the carrier and caveolin-3 is reduced. The data suggests that caveolin-3-containing structures are relevant for Ca^2+^ handling in cardiac cells (Camors et al., 2006). The involvement of caveolae in different cardiovascular diseases makes caveolin-based therapeutic approaches an attractive possibility to combat myocardial ischemia, heart failure and pulmonary hypertension (see Fridolfsson and Patel, 2013).

Cumulative evidence in the last decade has shown that key proteins in cardiomyocyte function interact with caveolins. Thus, caveolins not only participate in membrane positioning but also in ion channel regulation. Many ion transporters interact with caveolins and/or are located in caveolae (see Balijepalli and Kamp, 2008, for review). The human inward rectifying voltage-gated HERG K^+^ channel (Trudeau et al., 1995) interacts with caveolin-1 and reduces its activity when caveolin is up-regulated (Lin et al., 2008). Cell surface expression and degradation of HERG is also controlled by caveolin-3 via a complex of these two proteins with the Nedd4-2 ubiquitin ligase (Guo et al., 2012) and via dynamin-mediated endocytosis (Massaeli et al., 2010). Caveolin-3 interacts with another K^+^ inward rectifying channel (Kir2.1) whose current densities are affected by mutations in caveolin-3 (Vaidyanathan et al., 2013). Mutations in the cardiac hNa(v)1.5 channel lead to cardiac phenotypic manifestations; interestingly, caveolin-3 mutations identified in patients with inherited long-QT syndrome result in enhanced currents via this specific sodium channel (Vatta et al., 2006, see Wilde and Brugada, 2011, for review). Mutations in the caveolin-3 gene are also related to sudden infant death syndrome, with higher risk in carriers with further mutations in the hNa(v)1.5 gene (Arnestad et al., 2007; Cronk et al., 2007). Finally, caveolae may regulate ion handling by recruiting ion transporters and regulatory molecules (Yarbrough et al., 2002; Shibata et al., 2006; Palygin et al., 2008). A computer-based investigation supports the possibility that accumulation of ions in caveolae may lead to delayed-repolarization-induced arrhythmias (Besse et al., 2011). It is also of interest that caveolin-3 may be linked to and alters the function of hyperpolarization-activated cyclic nucleotide-gated channel 4, which regulates cardiac pacemaker activity (Ye et al., 2008). Balijepalli and Kamp (2008) provide a detailed account of the role of caveolae in arrhythmogenesis.

## Caveolae and neurological diseases

As neurological diseases are very diverse we present below a few examples of results that support a link between caveolins/caveolae and the two most prominent neurodegenerative disorders in developed countries: Parkinson's and Alzheimer's diseases.

α-synuclein is a protein that often accumulates in the brain of Parkinson's disease patients (Polymeropoulos et al., 1997; Arawaka et al., 1998; Takeda et al., 1998). α-synuclein seems to cause neurodegeneration by interacting with signaling proteins and/or altering receptor-mediated signaling pathways. Examples of proteins targeted by α-synuclein are protein kinase C, extracellular signal-regulated kinase (ERK) and phospholipase D (Ostrerova et al., 1999; Iwata et al., 2001; Ahn et al., 2002). Biochemical studies using α-synuclein-overexpressing human neuroblastoma cells show a correlation between altered ERK signaling and deregulation of caveolin-1 expression (Hashimoto et al., 2003). In parkinsonian patients but not in controls, six homozygous haplotypes of the caveolin-1 gene have been identified Darvish et al. (2013). Furthermore, the leucine-rich repeat kinase 2 (LRRK2) is located in the neck of caveolae in a human cell model (Alegre-Abarrategui et al., 2009). Mutations in the gene of this kinase are linked to Parkinson's disease (Di Fonzo et al., 2005; Gilks et al., 2005). Overall, the results establish a link between caveolin-1 and molecular hallmarks of Parkinson's disease.

Alzheimer's disease has two pathological hallmarks: intracellular neurofibrillary tangles, made up of aberrantly phosphorylated tau protein and plaques, made up of amyloidogenic (aberrant) processing of the amyloid precursor protein (APP), that leads to Aß peptide. The enzyme that cleaves APP to give Aß (BACE-1) physically associates to lipid-raft proteins (Hattori et al., 2006). Caveolin-3 is up-regulated in glial cells surrounding plaques and enhances the amyloidogenic route of APP processing (Nishiyama et al., 1999). Furthermore, caveolins physically associate to presenilins (Nishiyama et al., 1999) that are also relevant molecules in the pathophysiology of Alzheimer's disease. Therefore, caveolae were suspected to be involved in processing of APP, a transmembrane protein requiring integrity of cell surface mechanisms for a correct physiological function. The first indication for this view came in 1998 from the identification of the Aß peptide in detergent-insoluble compartments (Lee et al., 1998). More recently caveolin-1 knockout mice are considered a non-mutational model for Alzheimer's disease based on the accelerated aging and neurodegeneration phenotype (Head et al., 2010). The mice displayed astrogliosis and increased Aβ and hyperphosphorylated tau and, noteworthy, expression of caveolin-1 in neurons from the animals led to a significant decrease in Aβ expression (Head et al., 2010).

## Therapeutic possibilities: targeting caveolin expression

Caveolins/caveolae are now considered as therapeutic targets for a variety of diseases. Inhibitors are more frequent than activators as drugs targeting enzymes. Drugs targeting receptors are more often antagonists (blockers) than agonists (activators). Matters in the case of caveolins/caveolae are not as straightforward as caveolins have neither orthosteric binding sites for function regulation nor regulatory sites. To our knowledge, no small molecule targeting caveolins has been developed. Difficulties in targeting this type of membrane proteins likely underlie this lack of drug development and limit the use of caveolins as drug targets. One way to circumvent this issue is the use of anti-caveolin antibodies (Oh and Schnitzer, 1999; Gumbleton et al., 2003). Another limitation is the absence of suitable readouts for caveolin action. In sharp contrast there are highly suitable and high throughput possibilities for both enzymes and receptors. A significant alternative intervention to either increase or decrease caveolin expression would be gene or cell therapy, direct targeting of caveolin using antisense and siRNA approaches, modulation of cellular cholesterol levels or caveolar lipid content and the use of inhibitory peptides derived from caveolin scaffolding domains.

Regulation of cholesterol levels or caveolar lipid content may thus be attempted for therapeutic purposes. The use of statins such as 3-hydroxy-3-methylglutaryl-coenzyme A (HMG-CoA) reductase inhibitors decrease caveolin-1 levels (Kusama et al., 2001), which can involve blockade of the production of mevalonate, an intermediate in isoprenoid and cholesterol synthesis (Kirschmeier et al., 2001). Simvastatin may also alter lipid raft composition (Zhuang et al., 2005).

Despite the fact that gene therapy has not delivered the promised results, new viral vectors may be suitable to target caveolae with efficacy in the affected tissue. This would prevent side effects due to indiscriminate targeting of multiple tissues. Viruses can use caveolae-dependent or –independent mechanisms to enter the cell. One may take advantage of this fact to design therapeutic strategies using viral vectors that target caveolae (see next section). Analogously, polymeric structures may target caveolae and interfere with caveolae-mediated physiological or pathological actions. As an example, polysorbitol-based transporter delivery of small interfering RNA use caveolae for cell entry (Islam et al., 2012). Dendrimers successful in delivering genes via caveolae (Huang et al., 2014) merit attention for either enhancing or depressing caveolae-mediated events. Similarly, complexes formed by protamine, dextran, and solid lipid nanoparticles may target caveolae after intravenous administration to mice (Delgado et al., 2012).

## Therapeutic possibilities: from cell therapy to blockade of caveolae-mediated traffic and targeting of caveolin-protein/receptor interactions

Cell therapy is providing new hopes for a variety of diseases. Cell therapy using autologous stem cells engineered to express specific caveolins may be considered a further possibility to colonize tissues with caveolins/caveolae-related pathologies. To colonize lungs, Ghaedi et al. (2013) have been successful in developing caveolin-1 expressing alveolar epithelial cells from human induced pluripotent stem (iPS) cells. Similarly, other iPS-derived cells may have the potential to colonize endothelium in different tissues or, alternatively, iPS cells may differentiate into neurons (Chung et al., 2013) or, eventually into cardiomyocytes. To increase safety of cell therapy, generation of tumor-free iPS cell has been recently possible (Phillips, 2014; Zhang et al., 2014).

Caveolins/caveolae may be indirectly targeted by blocking membrane fusion events related to endocytosis. Dynasore, an inhibitor of the GTPase activity of dynamin may prevent the entry into cells of papilloma viruses that require caveolae- and clathrin-mediated endocytosis (Abban et al., 2008). Paramyxoviruses (Sánchez-Felipe et al., 2014) and neurotropic viruses such as Japanese encephalitis virus (Zhu et al., 2012) also use a caveolae and dynamin-dependent mechanism to enter into cells. Therefore, these viruses constitute a basis to design caveolae-targeting vectors.

Skeletal muscle expressing the Pro104Leu mutant of caveolin-3 leads to atrophy, and mice with this mutation serve as a model of limb-girdle muscular dystrophy 1C (Minetti et al., 1998; Galbiati et al., 2000; Hagiwara et al., 2000). The transforming growth factor type I β receptor kinase inhibitor, Ki26894, is able to restore both *in vitro* muscle cell deficiencies and muscle atrophy and weakness displayed by mutant mice (Ohsawa et al., 2012). A similar intervention could be envisaged for restoring cardiomyocyte function in patients with cardiac atrophy.

A further possibility is to modulate proteins located in caveolae to activate caveolae-mediated restorative events. Members of the G-protein-coupled receptor (GPCR) superfamily are a relevant example. GPCRs are targets of approximately 40% of compounds used in human therapy. Agonist and/or antagonist modulation of their activity can lead to increases or decreases in the expression levels of caveolins. In addition, it seems possible that GPCRs can form heteroreceptor complexes with caveolins. Interactions may modify the pharmacological properties in turn making possible the design of compounds selective for GPCR protomers present in such complexes. Examples of the direct link between caveolae and GPCRs are given in the next section.

Many proteins and receptors (e.g., GPCR) contain putative caveolin binding domains (Couet et al., 1997). For instance, caveolin-1 interacting proteins contain the canonical caveolin-1 binding domain, ϕXϕXXXXϕ or ϕXXXXϕXXϕ (where ϕ = Trp, Phe or Tyr). In some pathologies, reducing the ability of caveolins to couple to the signaling machinery at the inner plasma membrane may result in an efficacious intervention. For instance, the introduction of synthetic caveolin-scaffolding-domain peptides into cells may inhibit caveolin-protein interactions. In support of this, internalization of the caveolin scaffolding domain may be achieved by fusion of the domain with a 16-amino acid peptide of the *Drosophila antennapedia* homeodomain. By this approach platelet activating-factor-induced NO production and microvasculature permeability was reduced in tumor bearing animals (Zhu et al., 2004). It should be noted that neovascularization is required in initial steps of metastatic colonization of tissues and that caveolin-1 regulates metastasis in bladder cancer (Thomas et al., 2011).

Use of indirect caveolin-modulating strategies may also be effective against cardiovascular and neurological diseases. As detailed in the previous and the following sections, the bi-directional relationship that caveolins have with a number of interacting proteins and receptors could be exploited to re-expressing or targeting caveolins for up- and down-regulation. Examples would be targeting some GPCRs. The use of GPCR-selective agonist or antagonist, many of which are in current clinical use, may also affect directly (via receptor-caveolin interactions) or indirectly (via second messenger and signal cascade activation, e.g., MAPK) caveolin expression levels. Via activation of some GPCRs we could control or re-program caveolin expression levels to explore therapeutic outcomes in heart and brain.

## Caveolae and GPCRs

**Figure 1:**
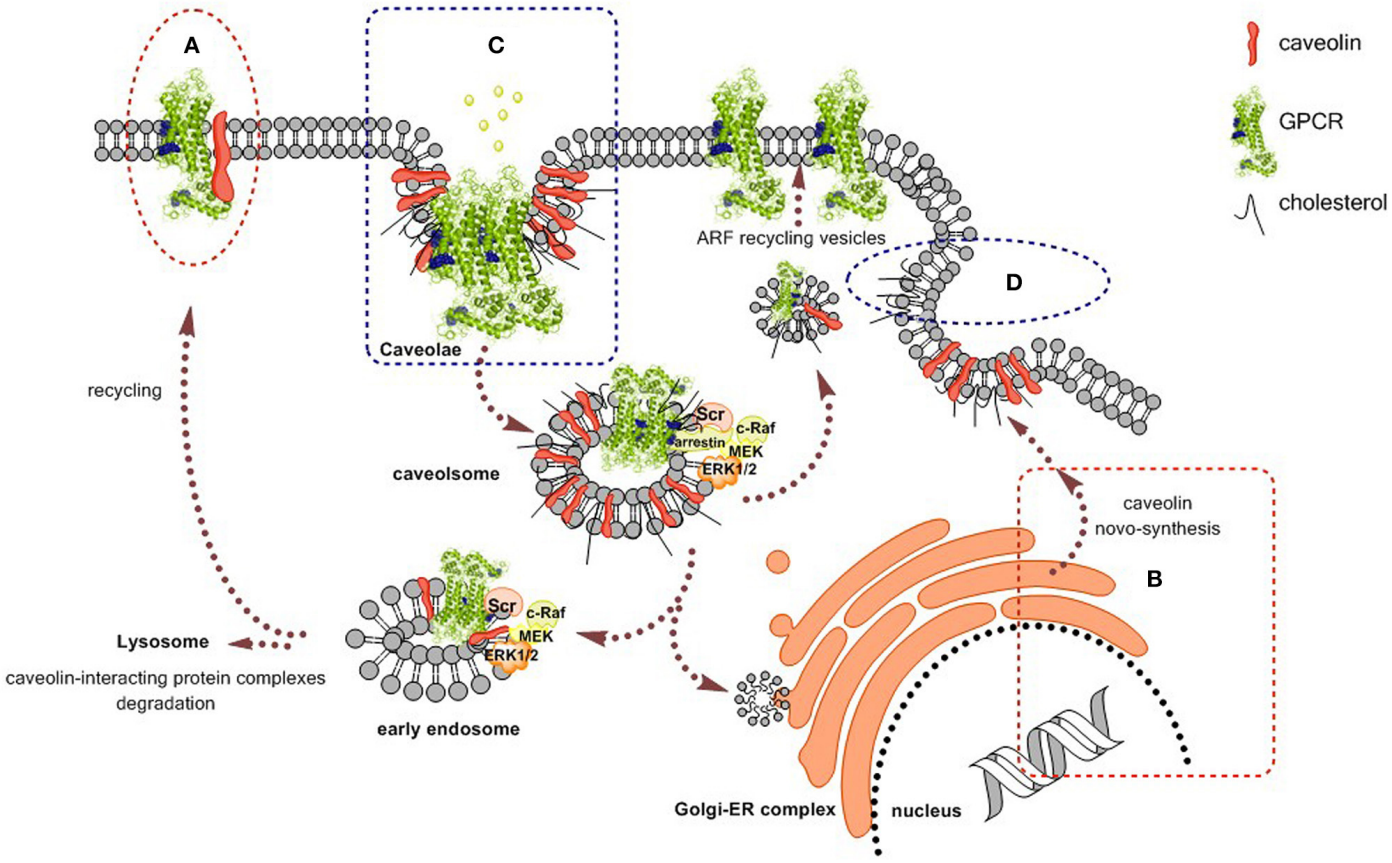
**Scheme of caveolins/caveolae participation in the cell biology of GPCRs**. Caveolins/caveolae regulate agonist binding and signaling and GPCR traffic. Some interventions with therapeutic potential are: **(A)** targeting caveolin-binding domains in the GPCR. **(B)** Regulating synthesis and expression of caveolins by means of cell therapy or small interfering RNA. **(C)** Targeting receptors to regulate caveolae-dependent endocytosis. **(D)** Regulation of cholesterol levels, for instance by using statins.

Cumulative evidence points to caveolae and caveolins as important regulators of GPCR traffic and function thus raising therapeutic potential in targeting caveolae or GPCRs in caveolae (see Figure 1). Caveolins form homo-oligomers (Monier et al., 1995; Sargiacomo et al., 1995) and interact with G proteins (Li et al., 1995). GPCRs are not homogeneously distributed on the cell surface and a significant amount of receptors are in caveolae (Ginés et al., 2001). GPCRs may even interact with caveolins (Burgueño et al., 2003, 2004). Even important elements in GPCR-mediated signaling such as G protein-coupled receptor kinases (GRKs) have binding motives for caveolins and the interaction regulates GRK function (Carman et al., 1999). Depending on cell type, and probably on caveolin subtype, GPCR agonists may enrich receptors in caveolae or do the opposite. In fact, agonist-induced activation of adenosine receptors may recruit them into caveolae for caveolae-mediated internalization (Ginés et al., 2001; Escriche et al., 2003). In contrast, in cardiomyocytes, these adenosine receptors are enriched in caveolae until activation leads to translocation out of caveolae (Lasley et al., 2000; Lasley and Smart, 2001). This differential behavior may be taken into account when targeting caveolae via GPCRs.

Specific G-protein-related signaling components are enriched in lipid rafts/caveolae meaning that these structures affect G-protein-coupling efficacy and signaling selectivity (see Chini and Parenti, 2004 and Insel et al., 2005, for review). An exhaustive review of the reports linking caveolins/caveolae to the biology of GPCRs is out of the scope of the present perspective article. Some few examples will, however, be provided to give a hint of the relevant connections between the receptors and caveolae. Localization of the α_1A_ adrenergic GPCR in lipid rafts restricts their conformation and basal activity while allowing a substantial coupling to the G protein and a robust signaling upon agonist activation (Lei et al., 2009). Caveolin-2 participates in receptor signaling even in a simple system constituted by human embryonic kidney (HEK-293) cells expressing a GPCR (D_1_) for the neurotransmitter dopamine (Yu et al., 2004). Caveolin is also involved in agonist-induced recruitment and internalization of a GPCR for the regulatory molecule adenosine (subtype 1, A_1_ receptor) (Ginés et al., 2001). In fact, Escriche et al. (2003) provided morphological evidence of caveolae-mediated internalization, endosomal sorting and A_1_ receptor recycling. Very relevant for caveolae-based drug discovery, Klaasse et al. (2005) reported small-molecule allosteric modulators of adenosine A_1_ GPCRs that affect internalization of the receptor. Adenosine deaminase, also interacts with A_1_ GPCRs, enhances signaling and appears to be an allosteric modulator of caveolae-mediated receptor internalization (Ginés et al., 2001). Caveolae disruption by cholesterol depletion alters the regulation by adenosine subtype 2 (A_2A_) of anion secretion in epithelial cells (Lam et al., 2009). Adenosine A_2A_ GPCRs are up-regulated in atrial fibrillation and their blockade results in restoring the abnormal calcium handling in cardiomyocytes from patients (Hove-Madsen et al., 2006; Llach et al., 2011). Adenosine A_2A_ GPCRs may also form homodimers or heteromers with other receptors. The complexes are unique entities with specific signaling properties (Hillion et al., 2002; Canals et al., 2004). Therefore, differential expression of A_2A_ GPCR monomer/homomers/heteromers in membrane micro-domains is an interesting possibility for altered functional properties that should be further explored. Endothelin subtype B receptor in endothelial cells is mainly present in caveolae and its activation by endothelin leads to rapid caveolae-dependent internalization. It is likely that activation of such receptors present in caveolae leads to rapid caveolae-mediated trafficking (Oh et al., 2012).

The selective advantage of reperfusion after ischemic injury to minimize the consequences of a second ischemic episode has been known for long (see Yellon et al., 1998 for review). From the finding that the increase in extracellular adenosine was helpful in anoxic heart conditions (Jacob and Berne, 1961; Ely and Berne, 1992) several studies have shown that adenosine (Kitakaze et al., 1993; Pelleg, 1993) and other compounds acting on GPCRs (Avkiran and Haworth, 2003; Minguet et al., 2012; Dragasis et al., 2013) regulate heart-ischemia-reperfusion and preconditioning. As reviewed by Stary et al. (2012) caveolins are necessary for ischemic preconditioning. Targeting for instance opioid receptors may help in enhancing the reperfusion benefits. Opioid-induced preconditioning alters the architecture of the myocyte and increases the number of caveolae (Tsutsumi et al., 2010; Stary et al., 2012). The use of knockout animals for caveolin-3 has shown that this protein is essential for *in vivo* opioid-induced preconditioning (Tsutsumi et al., 2010).

Future work will help on understanding how caveolin-protein and caveolin-GPCR interactions may help in combating cardiovascular and neurological diseases.

### Conflict of interest statement

The authors declare that the research was conducted in the absence of any commercial or financial relationships that could be construed as a potential conflict of interest.

## Abbreviations

GPCR: G-protein-coupled receptor
GRK: G protein-coupled receptor kinases.
